# Health Tract, No. 300

**Published:** 1867-08

**Authors:** 


HEALTH TRACT, No. 300.

### THE BEST RECOMMENDATION.

YOUNG men crowd to the great cities to seek their fortunes. With a consciousness of their own integrity they have an abiding confidence of success, but on applying first to one and then another but vainly, their eyes are gradually opened to the unwelcome fact that they must return whence they came, wondering why they can not " get in " somewhere, not knowing that for every vacancy a hundred quite as needy stand ready to fill it; their surprise is the greater, in that they had letters from General Smith, Governor Brown, Lawyer White and Parson Thomas.

Real business men, shrewd and keen-sighted, care very little about letters of recommendation from anybody, knowing that human nature is very accommodating in giving what costs nothing more than writing a few well expressed sentences. They know that truth lies in things, not words; in what they see rather than in what they hear. A youth would not get a clerkship with the recommendation of every governor in the nation, if he entered a counting-room with a cigar in his mouth, a cane in his hand and a diamond ring on his finger. A blundering swaggerer would wear out a dozen pair of boots on hard city pavements and yet fail to get wages from any prudent business man. Often the money of these young employment-seekers is exhausted before they know it, and before " remittances " come from hard working hands and affectionate hearts " at home," they have been tempted to drown sad thoughts at the theatre, the dance-house and the brothel, and all is lost. The most common, and the greatest mistake is in estimating their services entirely too high, considering the risks; not too high if they would be as faithful as they think they would, forgetting that the employer has no means of knowing their integrity. He must first prove that to the employer's entire satisfaction, and for the opportunity of doing it he ought to be glad for the chance of working for nothing but his board; and just as soon as he has shown himself to be prompt, faithful and energetic, his good qualities will be appreciated, his rise will be speedy and his ultimate success sure.

Business men often form determinations involving thousands in value from apparently trifling incidents. A young man seeking employment went to one of our large cities, and, on enquiring at a certain counting-room if they wished a clerk, was told that they did not. In turning over his carpet bag to find his letters, a book rolled out on the floor. " What book is that ? " said the merchant. " It is the Bible, sir," was the reply. " And what are you going to do with that book in New York ? " The lad looked seriously into the merchant's face, and replied, " I promised my mother I would read it every day, and I shall do it," and burst into tears. The merchant immediately engaged his services, and in due time, he became a partner in the firm, one of the most respectable in the city.

A young man once asked a city merchant to sell him some goods on short credit; his recommendations were very favorable, but the times were uncertain, many were failing, and the credit was refused; as he was slowly leaving the store, greatly depressed at the disappointment, he stooped to pick up a pin, and carefully stuck it in his coat collar. The merchant noticed it, called him back and told him he could have what goods he wanted, " for I see you are careful and economical of small things — such men can be safely trusted." — A young man purchased a bundle of goods from Stephen Girard: as he was about to shoulder them at the door, Mr. G. said to him, " Why don't you call that dray ? " " O," said the youth, " I can carry it home myself and save the cartage." Mr. G. was so well pleased with the reply that he took the youth into his confidence and befriended him as long as he lived.

